# Nigral Iron Deposition Is Associated With Levodopa-Induced Dyskinesia in Parkinson’s Disease

**DOI:** 10.3389/fnins.2021.647168

**Published:** 2021-03-22

**Authors:** Tianbin Song, Jiping Li, Shanshan Mei, Xiaofei Jia, Hongwei Yang, Yongquan Ye, Jianmin Yuan, Yuqing Zhang, Jie Lu

**Affiliations:** ^1^Department of Radiology and Nuclear Medicine, Xuanwu Hospital, Capital Medical University, Beijing, China; ^2^Beijing Key Laboratory of Magnetic Resonance Imaging and Brain Informatics, Beijing, China; ^3^Beijing Institute of Functional Neurosurgery, Xuanwu Hospital, Capital Medical University, Beijing, China; ^4^Department of Neurology, Xuanwu Hospital, Capital Medical University, Beijing, China; ^5^UIH America, Inc., Houston, TX, United States; ^6^Central Research Institute, UIH Group, Shanghai, China

**Keywords:** Parkinsion’s disease, dyskinesia, levodopa, quantitative susceptibility mapping, substantia nigra

## Abstract

**Objective:**

To investigate iron deposition in the substantia nigra (SN) of Parkinson’s disease (PD) patients associated with levodopa-induced dyskinesia (LID).

**Methods:**

Seventeen PD patients with LID, 17 PD patients without LID, and 16 healthy controls were recruited for this study. The mean QSM values of the whole, left, and right SN were compared among the three groups. A multivariate logistic regression model was constructed to determine the factors associated with increased risk of LID. The receiver operating characteristic curve of the QSM value of SN in discriminating PD with and without LID was evaluated.

**Results:**

The mean QSM values of the whole and right SN in the PD with LID were higher than those in the PD without LID (^∗^*P* = 0.03, ^∗^*P* = 0.03). Multivariate logistic regression analysis revealed that the QSM value of whole, left, or right SN was a predictor of the development of LID (^∗^*P* = 0.03, ^∗^*P* = 0.04, and ^∗^*P* = 0.04). The predictive accuracy of LID in adding the QSM value of the whole, left, and right SN to LID-related clinical risk factors was 70.6, 64.7, and 67.6%, respectively. The QSM cutoff values between PD with and without LID of the whole, left, and right SN were 148.3, 165.4, and 152.7 ppb, respectively.

**Conclusion:**

This study provides the evidence of higher iron deposition in the SN of PD patients with LID than those without LID, suggesting that the QSM value of the SN may be a potential early diagnostic neuroimaging biomarker for LID.

## Introduction

Parkinson’s disease (PD) is a progressive movement disorder caused primarily by the loss of nigrostriatal neurons and depletion of dopamine (Thomas and Beal, 2007). Dopamine replacement therapeutics, such as levodopa, relieve the symptoms of PD but can lead to a motor complication known as levodopa-induced dyskinesia (LID), which is a major cause of disability in PD patients (Pahwa et al., 2019). LID is observed in approximately 90% of PD patients after 10 years of levodopa treatment (Manson et al., 2012).

Iron is essential for normal brain functions, but iron overload has been highly implicated in the pathology and pathogenesis of PD. Biochemical and neuroimaging studies have shown increased iron deposition in the nigrostriatal pathway in PD patients, specifically in the substantia nigra (SN) (Rouault, 2013; Ward et al., 2014; Du et al., 2016; Acosta-Cabronero et al., 2017). Iron deposition in the SN in PD patients, determined *via* quantitative susceptibility mapping (QSM) of iron-sensitive magnetic resonance imaging (MRI) (Homayoon et al., 2019; Sun et al., 2020), was significantly higher than that in healthy people and in patients with idiopathic rapid eye movement sleep behavior disorder, dystonia, and essential tremor. Iron deposition also contributes to the clinical motor symptoms of PD (Martin et al., 2008; He et al., 2015). An existing study also showed that the R2^∗^ value of SN in PD patients with LID was higher than those without LID (Lewis et al., 2013). However, the association between the iron content of SN measured by QSM and the LID is still unclear.

Therefore, this study aimed at using QSM to investigate the iron deposition difference in SN between PD patients with and without LID and to determine whether iron deposition in the SN is a potential neuroimaging biomarker of LID event.

## Materials and Methods

### Subjects

Thirty-four PD patients (17 with LID and 17 without LID) who were prepared to undergo deep brain stimulation surgery were recruited from the functional neurosurgery department of the Xuanwu Hospital of Capital Medical University. All PD patients were evaluated using the Movement Disorder Society’s revision of the Unified Parkinson’s Disease Rating Scale (MDS-UPDRS) Part III for motor symptoms in the medication-off state (Postuma et al., 2015), after at least a 12-h overnight withdrawal from dopaminergic medication, the Unified Dyskinesia Rating Scale (UDysRS) for dyskinesia in on and off state, and UPDRS Part IV for severity of motor fluctuation. All PD patients belong to akinetic rigid subtype. All assessments were performed by a single trained neurologist (10 years). The inclusion criteria for PD patients were as follows: (1) meeting the MDS clinical diagnostic criteria of PD (Postuma et al., 2015), (2) had undergone levodopa treatment for at least 6 months and stable medication dose for at least 4 weeks, and (3) with no history of cerebrovascular disease, seizures, brain surgery, and psychiatric disorders. PD patients with evident cerebral lesions on MRI structural images and evident motion artifacts in QSM images were excluded. PD patients with a score of MDS-UPDRS Part IV item 33 ≥ 1 were included in the LID group, and the rest with a score of zero were included in the PD without LID group. Sixteen age- and sex-matched healthy controls (HCs) were recruited from the local community. All of the HCs had normal movement function and no neurological or psychiatric diseases (Table 1). All experiments in this study were approved by the Institutional Review Board of Xuanwu Hospital. All participants signed informed consent forms prior to the experiment.

**TABLE 1:** Demographic and clinical characteristics of the study participants and mean QSM values in whole, left, and right SN.

	PD with LID	PD without LID	HCs	ANOVA	*P* value (*post hoc*)		
	(*n* = 17)	(*n* = 17)	(*n* = 16)	*P* value	LID vs without LID	LID vs HCs	Without LID vs HCs
Age (years old)	63.88 ± 4.64	65.2 ± 7.18	61.06 ± 4.42	0.11	—	—	—
Age of onset	55 ± 5.21	54.88 ± 10.85	—	0.94	—	—	—
Gender (female)	17 (12)	17 (12)	16 (10)	0.85	—	—	—
BMI (kg/m^2^)	23.98 ± 0.90	23.53 ± 1.21	23.75 ± 2.43	0.94	—	—	—
Onset of motor symptom	10L/7R	7L/10R	—	0.49	—	—	—
Disease duration (years)	9 ± 2.52	8.53 ± 3.41	—	0.65	—	—	—
H-Y stage	2.85 ± 0.63	2.79 ± 0.64	—	0.79	—	—	—
Duration of levodopa therapy (years)	7.53 ± 2.15	7.06 ± 3.61	—	0.65	—	—	—
MDS-UPDRS III score	50.29 ± 10.43	63.24 ± 10.78	—	0.04*	—	—	—
UPDRS IV score	9.9 ± 2.1	5.5 ± 3.6	—	<0.01**	—	—	—
LEDD (mg/day)	1,099 ± 581	814 ± 359	—	0.09	—	—	—
LEDD of levodopa (mg/day)	540 ± 164	509 ± 137	—	0.56	—	—	—
UDysRS total score	23.59 ± 9.86	—	—	—	—	—	—
UDysRS score on off state	6.3 ± 4.2 (10/17)	—	—	—	—	—	—
LID duration (months)	25.52 ± 14.40	—	—	—	—	—	—
Whole SN (ppb)	162.36 ± 26.85	140.25 ± 26.85	113.23 ± 21.5	<0.01**	0.03*	<0.01**	<0.01**
Left SN (ppb)	159.17 ± 29.68	139.47 ± 29.68	108.07 ± 24.43	<0.01**	0.09	<0.01**	<0.01**
Right SN (ppb)	165.55 ± 28.51	141.03 ± 28.518	118.40 ± 21.23	<0.01**	0.03*	<0.01**	0.02*

The values presented are the number of patients or mean ± standard deviation. LID, levodopa-induced dyskinesia; MMSE, Mini-Mental State Exam; PD, Parkinson’s disease; H-Y stage, Hoehn-Yahr stage; MDS-UPDRS III, Part III of Movement Disorder Society’s Revision of the Unified Parkinson’s Disease Rating Scale (MDS-UPDRS); LEDD, levodopa equivalent daily dose; UDysRS, Unified Dyskinesia Rating Scale; HCs, healthy controls. ppb, parts per billion; L, left; R, right; BMI, body mass index. *P < 0.05 and **P < 0.01 were considered statistically significant.

### MRI Data Acquisition

Magnetic resonance imaging data acquisition was performed using a hybrid 3.0-T PET/MR scanner (uPMR790, UIH, Shanghai, China) with a 24-channel head/neck coil. A three-dimensional (3D) multi-echo gradient-echo (GRE) sequence was used for QSM data acquisition with the following parameters: FA = 15°, voxel size = 1 mm × 1 mm × 2 mm (interpreted as 1 mm^3^), repetition time = 29 ms, six echo times = 3.1/6.4/9.7/13.0/16.3/19.6 ms, and bandwidth = 500 Hz/px, acquisition matrix: 256 × 256, number of slices: 68, slice orientation: F-H, parallel imaging, acceleration factor: 2, and monopolar readout gradients were used with a total scan time of 5 min and 41 s. T2-weighted imaging (T2WI), diffusion-weighted imaging, and 3D T1-weighted imaging (T1WI) data were also collected from all participants. During data acquisition, the participant’s head was stabilized with foam pads on both sides to reduce motion. All PD patients were scanned during their off-state condition (12 h after last medication).

### QSM Image Processing

The multi-echo GRE data were used for the QSM calculation. The B0 field map was extracted using the multidimensional integration method, unwrapped of aliased phases using seed prioritized unwrapping (SPUN) (Ye et al., 2019), and the background field was removed using vSHARP. QSM was obtained by solving the L1 regularization problem using the precondition conjugate gradient method (Bilgic et al., 2014), with an extra term for streaking artifact suppression (Li et al., 2015) and dynamic Bayesian regularization (De Rochefort et al., 2010). We used CSF as the reference tissue, as suggested in the review article (Haacke et al., 2015). To draw the CSF reference region of interest (ROI), first the short TE magnitude images of the multi-echo GRE was selected, which showed a clear boundary of the CSF. Then, we manually drew the ROI on this short TE magnitude image, placed it centrally in the cerebrospinal fluid of the lateral ventricles and avoided other non-CSF contents. Then, this ROI was directly copied to the QSM image of the same slice, which was calculated from the same data and thus was strictly aligned with each other. For each case, three or more CSF ROIs were determined, and the mean QSM value from these ROIs was used as the final reference.

### Regions of Interest

**FIGURE 1:**
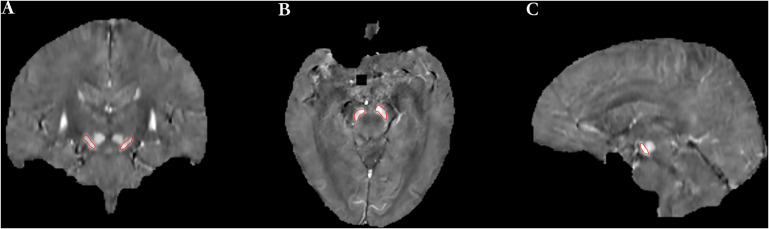
The definition of regions of interest (ROIs) included the left and right SN [**(A)** coronal image, **(B)** axial image, and **(C)** sagittal image].

Reference region of interests were manually drawn on left and right SN as shown in Figure 1 and were used to extract QSM values. When drawing ROIs on bilateral SNs, the first and last slices of SN were discarded, and those remaining consecutive slices showing portions of the SN with clear and visible boundaries were used. As a result, generally four to five slices were measured for each case. To assess the inter-rater reliability of the ROIs, two radiologists were instructed to draw the ROIs independently. Mean QSM values of the left and right SN were measured, and the average of the two was used as the mean QSM value of the whole SN.

### Statistical Analysis

The intraclass correlation coefficient (ICC) was used to evaluate the inter-rater reliability of the measured QSM value of left and right SN. An ICC value of over 0.81 was considered to indicate excellent agreement, 0.61–0.80 good agreement, 0.41–0.60 moderate agreement, 0.21–0.40 fair agreement, and less than 0.20 poor agreement. ICC values were calculated by using the International Business Machines Statistical Package for the Social Sciences (SPSS) version 17.0 software.

The demographic characteristics were compared among the three groups, and the chi-squared test was applied for sex, onset side of motor symptom, and analysis of variance (ANOVA) analysis for age and age of onset. Two-tailed *‘* tests were performed to compare disease duration, duration of levodopa therapy, H-Y stage, MDS-UPDRS III score, MDS-UPDRS IV score, levodopa equivalent daily dose (LEDD), and LEDD of levodopa between PD patients with and without LID. The mean QSM values of the whole SN, left SN, and right SN were compared by using ANOVA among the three groups, respectively. Statistical analysis was performed using GraphPad Prism 7.0.

Multiple variables were included in a multivariate logistic regression model analysis to determine the independent risk factors associated with LID, including iron content of the whole SN, sex, body mass index (BMI), disease duration, duration of levodopa therapy, age of onset, and LEDD/LEDD of levodopa, as well as left and right SN, respectively. First, multivariate logistic regression model analysis was used to calculate the diagnostic accuracy rate of all clinical risk factors. Second, we added the iron content of SN into all these clinical risk factors to perform a multivariate logistic regression model analysis. The effect of the iron content of SN on the predictive accuracy rate of LID was evaluated. Statistical analysis was performed using the SPSS for Windows (version 17.0, SPSS Inc., Chicago, IL, United States).

The discriminative power of the QSM value on LID was evaluated in the whole, left, and right SN, respectively, by the receiver operating characteristic curve. Pearson’s correlation analysis was used to study the association between the QSM values of whole, left, and right SN and clinical assessments (UDysRS total score and LID duration) in the PD with LID group, respectively. Statistical analysis was performed using GraphPad Prism 7.0. *P* < 0.05 was considered a statistically significant different.

## Results

### Participants’ Characteristics

Demographic details are summarized in Table 1. There were no significant differences in almost all clinical and demographic data between the groups of PD patients with and without LID, or the HCs (*P* > 0.05), except for UPDRS III and IV scores between PD patients with and without LID (^∗^*P* = 0.04 < 0.05, and ^∗∗^*P* < 0.01). Also, no significant associations were observed between the QSM values of SN and UPDRS III scores in both PD groups (*P* > 0.05, *P* > 0.05). UPDRS III score of PD without LID was slightly higher than PD with LID in this study. UPDRS IV score of PD with LID was slightly higher than PD without LID in this study.

### Test–Retest Reliability Analysis

The test–retest reliability of the ROI drawn manually by two radiologists was evaluated by ICC analysis. The ICC value of consistency in the left and right SN between them was 0.95 and 0.97, respectively, indicating excellent intra-rater agreement.

### Comparison of the Mean QSM Value of SN Among Three Groups

**FIGURE 2:**
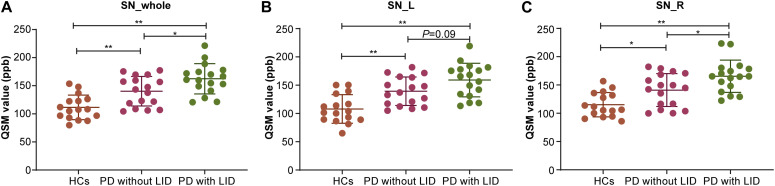
Compare mean quantitative susceptibility mapping (QSM) value of the whole, left, and right SN among the three groups. **(A)** Analysis of variance (ANOVA) analysis of QSM value in the whole SN (SN_whole): Parkinson’s disease (PD) with levodopa-induced dyskinesia (LID) > PD without LID > healthy controls (HCs) (*P_LID vs without LID_ < 0.05, **P_LID VS HCs_ < 0.01, **P_*withoutLID* VS HCs_ < 0.01). **(B)** ANOVA analysis of QSM value in the left SN (SN_L): PD with LID>HCs (**P_LID vs HCs_ < 0.01) and PD without LID<HCs (**P_without LID vs HCs_ < 0.01). **(C)** ANOVA analysis of QSM value in the right SN (SN_R): PD with LID<PD without LID < HCs (*P_*LID* VSwithout LID_ < 0.05, **P_LID vs HCs_ < 0.01, *P_*withoutLIDvsHCs*_ < 0.05).

The mean QSM values of the left and right SN in each group are shown in Table 1 and Figure 2. PD with LID had significantly higher QSM values in the right and whole SN than PD without LID (^∗^*P* = 0.03 < 0.05, and ^∗^*P* = 0.03 < 0.05) and HCs (^∗∗^*P* < 0.01 and ^∗∗^*P* < 0.01). PD without LID had a significantly higher QSM value in the left, right, and whole SN than that of HCs (^∗∗^*P* < 0.01, ^∗∗^*P* < 0.01, and ^∗^*P* = 0.02 < 0.05), but no significant difference was found in the left SN between PD with and without LID (*P* > 0.05). UPDRS IV score was added as a covariate to compare the difference between PD patients with LID and without LID group, significant difference in the QSM value of left SN was found between PD patients with LID and without LID groups (*t* = 6.131, ^∗^*P* = 0.02 < 0.05), there was no significant difference in the QSM value of whole SN but the *P* value is close to 0.05 between two groups (*t* = 3.902, *P* = 0.057 ≈ 0.05), and no significant difference in the QSM value of right SN was found between two groups (*t* = 2.690, *P* = 0.11 > 0.05).

### Multivariate Logistic Regression Analysis for LID Related Risk Factors

The predictive accuracy of LID to LID-related clinical risk factors, with added factors of the QSM value of whole and left and right SN, was 70.6, 64.7, and 67.6% respectively. Also, iron deposition of the whole, left, and right SN can be considered as an independent predictor of LID (^∗^*P* < 0.05, ^∗^*P* < 0.05, and ^∗^*P* < 0.05) (Tables 2, 3).

**TABLE 2:** Multivariate logistic regression analysis of risk factors correlated to LID event among 34 PD patients.

	Whole SN		Left SN		Right SN	
	OR (95% CI)	*P* value	OR (95% CI)	*P* value	OR (95% CI)	*P* value
QSM value	1.038 (1.003–1.074)	0.03*	1.033 (1.002–1.065)	0.04*	1.035 (1.002–1.070)	0.04*
Age of onset	0.993 (0.890–1.107)	0.90	0.993 (0.889–1.111)	0.91	0.988 (0.887–1.100)	0.83
Sex	1.828 (0.255–13.098)	0.55	1.741 (0.257–11.808)	0.57	2.009 (0.280–14.402)	0.49
BMI	1.028 (0.844–1.252)	0.78	1.013 (0.832–1.233)	0.90	1.046 (0.862–1.270)	0.65
Disease duration	1.032 (0.549–1.942)	0.92	1.105 (0.628–1.945)	0.73	1.004 (0.516–1.953)	0.99
Duration of levodopa therapy	0.916 (0.494–1.701)	0.78	0.888 (0.498–1.583)	0.69	0.924 (0.489–1.745)	0.81
LEDD	1.002 (1.000–1.004)	0.09	1.002 (1.000–1.004)	0.07	1.002 (1.000–1.004)	0.11

Data were analyzed using multivariate logistic regression analysis [adapted factors: quantitative susceptibility mapping value, age of onset, sex, disease duration (years), duration of levodopa therapy (years), and levodopa equivalent daily dose (mg/day), LEDD]. BMI, body mass index; OR, odds ratio; CI, confidence interval. *P < 0.05 was considered statistically significant.

**TABLE 3:** Multivariate logistic regression analysis of risk factors correlated to LID event among 34 PD patients.

	Whole SN		Left SN		Right SN	
	OR (95% CI)	*P* value	OR (95% CI)	*P* value	OR (95% CI)	*P* value
QSM value	1.038 (1.003–1.074)	0.03*	1.032 (1.002–1.064)	0.04*	1.036 (1.004–1.069)	0.03*
Age of onset	1.020 (0.921–1.130)	0.70	1.023 (0.922–1.134)	0.67	1.015 (0.917–1.123)	0.78
Sex	0.777 (0.134–4.491)	0.78	0.769 (0.139–4.249)	0.76	0.869 (0.153–4.945)	0.87
BMI	0.998 (0.814–1.224)	0.98	0.977 (0.799–1.194)	0.82	1.022 (0.835–1.251)	0.83
Disease duration	1.020 (0.921–1.130)	0.70	0.957 (0.586–1.563)	0.86	0.87 (0.488–1.551)	0.64
Duration of levodopa therapy	1.123 (0.659–1.912)	0.67	1.098 (0.665–1.813)	0.71	1.127 (0.649–1.956)	0.67
LEDD of levodopa	1.003 (0.997–1.008)	0.35	1.003 (0.998–1.008)	0.29	1.002 (0.997–1.007)	0.46

Data were analyzed using multivariate logistic regression analysis [adapted factors: quantitative susceptibility mapping value, age of onset, sex, disease duration (years), duration of levodopa therapy (years), and levodopa equivalent daily dose (mg/day), LEDD]. BMI, body mass index; OR, odds ratio; CI, confidence interval. *P < 0.05 was considered statistically significant.

### Receiver Operating Characteristic Curve Analysis for QSM Value in Discriminative Efficacy Among Three Groups

**FIGURE 3:**
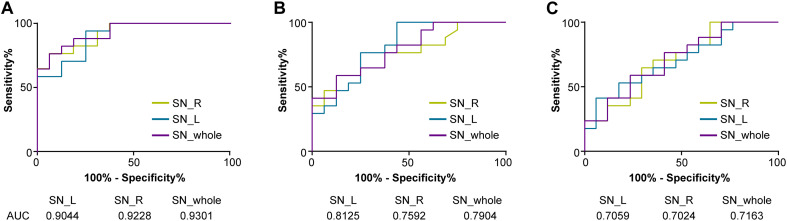
Receiver operating characteristic (ROC) curves showing the accuracy of QSM in differentiating between LID and HCs **(A)**, PD without LID and HCs **(B)**, and LID and PD without LID **(C)** in the SN (whole SN, left SN, and right SN, respectively). The diagnostic performance of the QSM value was defined by the area under the ROC curve (AUC). QSM, quantitative susceptibility mapping; PD, Parkinson’s disease (PD); LID, levodopa-induced dyskinesia; HCs, healthy controls; SN_whole, whole SN; SN_L, left SN; SN_R; right SN.

The discriminative power of the QSM value in the whole, left, and right SN among three groups is shown in Figure 3. The cutoff value of the whole SN QSM for discrimination between PD patients with LID and HCs was 127.3 ppb (accuracy, 0.9301; sensitivity, 0.8824; specificity, 0.8124) (^∗∗^*P* < 0.01). This QSM cutoff value was 117.7 ppb (accuracy, 0.9044; sensitivity, 0.9412; specificity, 0.75) (^∗∗^*P* < 0.01) and 146.9 ppb (accuracy, 0.9228; sensitivity, 0.7647; specificity, 0.9375) (^∗∗^*P* < 0.01) for left and right SN, respectively. Between PD without LID and HC groups, the QSM cutoff values were 137.2 ppb (accuracy, 0.7904; sensitivity, 0.5882; specificity, 0.875) (^∗∗^*P* < 0.01), 106.6 ppb (accuracy, 0.8125; sensitivity, 0.9412; specificity, 0.5625) (^∗∗^*P* < 0.01), and 115.3 ppb (accuracy, 0.7592; sensitivity, 0.7647; and specificity, 0.625) (^∗^*P* = 0.01 < 0.05) in the whole, left, and right SN, respectively. Between PD groups with and without LID, the QSM cutoff values were 148.3 ppb (accuracy, 0.7163; sensitivity, 0.7647; specificity, 0.5882) (^∗^*P* = 0.03 < 0.05), 165.4 ppb (accuracy, 0.7059; sensitivity, 0.5294; specificity, 0.8235) (^∗^*P* = 0.04 < 0.05), and 152.7 ppb (accuracy, 0.7024; sensitivity, 0.7059; specificity, 0.6471) (^∗^*P* = 0.04 < 0.05) in the whole, left, and right SN, respectively.

### Associations Between the QSM Value of the SN and UDysRS or LID Duration

No significant correlation was observed between the QSM values of the SN and UDysRS total scores (whole SN, *R* = −0.26 and *P* = 0.31 > 0.05; left SN, *R* = −0.21 and *P* = 0.42 > 0.05; right SN, *R* = −0.33 and *P* = 0.20 > 0.05, respectively) or between the QSM values of the SN and LID duration (whole SN, *R* = −0.21 and *P* = 0.43 > 0.05; left SN, *R* = 0.18 and *P* = 0.50 > 0.05; right SN, *R* = −0.20 and *P* = 0.43 > 0.05, respectively).

## Discussion

Iron deposition in the whole and right SN was significantly higher in PD patients with LID compared to PD patients without LID and HCs, but it was not associated with the severity of LID or LID duration. Our results also revealed that iron deposition of the whole, left, and right SN was an independent risk factor of LID among LID-related clinical risk factors of iron content of SN, sex, disease duration, duration of levodopa therapy, age at onset, LEDD, and LEDD of levodopa.

We should study the structure of SN, which highly expresses D1 and D2 receptors, though most studies focused on the striatum. Studies on dyskinetic rats revealed a relationship between the expression of abnormal involuntary movements and elevated level of extracellular GABA in SN (Mela et al., 2007), which suggests that GABA released from nigrostriatal neurons generates LID. Elevated released levels of GABA in SNr were induced by L-DOPA, reflecting hyperactivity of nigrostriatal pathway, which suggests that hyperactivity plays an important role in LID event (Cenci, 2007). D1 and D2 receptor blockaded in SNr reduced LID event that happened, which suggests that SN generates dyskinesia. L-DOPA can be converted to DA in SNr (Sarre et al., 1998), and L-DOPA administration leads to elevated level of extracellular DA in SNr and striatum of rats with dyskinesia (Lindgren et al., 2010; Mela et al., 2012).

Iron is an essential element involved in numerous neurobiological processes in the human brain (Jenner, 2008; Prashanth et al., 2011). However, iron can also be detrimental by causing oxidative stress *via* redox reactions, which can even lead to neuronal apoptosis in iron-overloaded cells (Cozzi et al., 2019). The accumulation of iron was confirmed to be related to the pathophysiology of PD, such as the loss of dopaminergic neurons in the SN and the presence of α-synuclein-rich Lewy bodies (Dexter et al., 1991; Bisaglia et al., 2014; Murakami et al., 2015). Previous studies have revealed that the iron content of the SN is increased in PD patients (Kordower et al., 2013; Guan et al., 2017). Excessive iron deposition contributes to the dysfunction of the nigrostriatal pathway, which is related to motor dysfunction in PD patients (Martin et al., 2008; He et al., 2015; Hare and Double, 2016), but previous studies have not reported the relationship between SN cell loss and LID event. Nigral cell degeneration leads to the function alterations of basal ganglia, which is a cause of dyskinesia, and the degeneration extent of SN may regulate the duration of drug exposure, which induces dyskinesia (Jenner, 2008).

The previous study showed that the R2^∗^ value of SN in PD with LID was higher than PD without LID (Lewis et al., 2013). However, it has been shown that QSM is more sensitive than R2^∗^ mapping in the detection of the increased iron levels of SN in PD patients (Deistung et al., 2013; Barbosa et al., 2015; Du et al., 2016). In the present study, we directly compared the QSM value of SN between PD with LID and without LID, and found that the QSM values of the whole and right SN of PD with LID was significantly higher than that of PD without LID. It demonstrates that excess iron deposition in the SN actually happens in PD patients with LID, which is an important factor that contributes to the LID event. We also found that iron depositions in the whole, left, and right SN of PD with the LID and without LID groups are both significantly higher than that of HCs, which demonstrated that iron deposition of SN in patients with PD is higher than that in HCs, which is consistent with a previous study (Du et al., 2016). However, we did not find that iron deposition in the left SN in PD with LID being higher than that of PD without LID, which was not reported by previous studies. No relationships between the R2^∗^ value of SN and levodopa dosage, UPDRS-III score, LEDD, and disease duration were reported in either PD with LID or without LID in a previous study (Du et al., 2016), nor did those clinical scores associated with QSM values of the whole, left, and right SN in either PD with LID or without LID.

Levodopa-induced dyskinesia is a common and potentially disabling complication of levodopa treatment in patients with PD. Evidence indicates that LID occurs in response to pulsatile dopaminergic stimulation together with the short half-life of levodopa (Espay et al., 2018). The younger age of onset, longer disease duration, higher levodopa dose, and longer duration of levodopa therapy are considered major clinical risk factors for LID (Zappia and Quattrone, 2008; Warren et al., 2013; Cilia et al., 2014; Perez-Lloret et al., 2017; Matarazzo et al., 2018; Picconi et al., 2018). Young PD patients had a higher risk of LID than PD patients with old age of onset (Kumar et al., 2005; Dhall and Kreitzman, 2016). We analyzed the effect of iron content of SN on all the above clinical risk factors and found that the QSM value of the whole SN showed high accuracy in discriminating PD with LID and HCs (whole SN, 0.9273; left SN, 0.9066; right SN, 0.91) and PD with LID and without LID (whole SN, 0.7163; left SN, 0.7059; right SN, 0.7024). The QSM cutoff value of the SN may help predict LID event in PD progression. We found that the QSM cutoff value of the whole SN, which was 127.3 ppb, can discriminate between PD with LID and HCs (sensitivity, 88.24%; specificity, 81.24%), and for the left and right SN, the cutoff values were 117.7 ppb (sensitivity, 94.12%; specificity, 75%) and 146.9 ppb (sensitivity, 76.47%; specificity, 93.75%), respectively. The QSM cutoff value of the whole SN, left SN, and right SN was 148.3 ppb (sensitivity, 76.47%; specificity, 58.82%), 165.4 ppb (sensitivity, 52.94%; specificity, 82.35%), and 152.7 ppb (sensitivity, 70.59%; specificity, 64.71%) in discrimination between PD with and without LID, respectively. These findings suggest that the QSM value of SN can well discriminate between PD patients with LID and without LID; this may suggest that PD patients with a low QSM value in the SN were less prone to develop LID.

We also investigated the correlations between the iron content of SN with UDysRS score and LID duration. No significant associations between them were found, suggesting that iron deposition in the SN is not associated with the severity and duration of LID.

Despite these strengths, the main limitation of our study was the small sample size. Larger cohorts will be further recruited to validate the results, and longitudinal prospective evaluation of changes of the iron deposition over time in LID patients is needed to be studied in the future. The correlation between the QSM value of the sub-regions of the SN, such as pars compacta and pars reticulata, and LID also needs to be further studied in the future. The correlation between the loss of DA neurons and iron deposition of SN in PD with LID should be studied in the future. The difference in the QSM value of SN among off period dystonia, peak dose dyskinesia, and diphasic dyskinesia of LID groups also needs to be further studied in the future.

## Conclusion

In conclusion, our study revealed that iron deposition in the SN was significantly higher in PD patients with LID than in those without LID by using the QSM method, but it was not associated with the severity of LID. Iron deposition in the SN measured by QSM sequence is a potential neuroimaging biomarker of LID event.

## Data Availability Statement

The raw data supporting the conclusions of this article will be made available by the authors, without undue reservation.

## Ethics Statement

The studies involving human participants were reviewed and approved by all experiments in this study were approved by the Institutional Review Board of Xuanwu Hospital. The patients/participants provided their written informed consent to participate in this study.

## Author Contributions

TS and JL: execution of the project, statistical analysis, interpretation of data, and first draft of the manuscript. SM: review and critique of statistical analysis. XJ and YY: statistical analysis. HY: performed MR scan and statistical analysis. JY: review and critique of statistical analysis. YZ: conception of the project and review and critique of the manuscript. JL: conception and organization of the project, review and critique of the manuscript. All authors contributed to the article and approved the submitted version.

## Conflict of Interest

YY was employed by the company UIH America. The remaining authors declare that the research was conducted in the absence of any commercial or financial relationships that could be construed as a potential conflict of interest.
